# Suppression of cytoplasmic incompatibility in the leaf-mining fly *Liriomyza sativae* with a nuclear *Wolbachia* insert

**DOI:** 10.1098/rsos.242137

**Published:** 2025-05-21

**Authors:** Yuta Ohata, Takafumi N. Sugimoto, Nicky Wybouw, Yohsuke Tagami

**Affiliations:** ^1^ Gifu University Faculty of Agriculture, Gifu University, Gifu, Japan; ^2^ Institute of Agrobiological Sciences, National Agriculture and Food Research Organization, Tsukuba, Ibaraki Prefecture, Japan; ^3^ Department of Biology, Ghent University, Ghent, Belgium; ^4^ Graduate School of Agriculture, Shizuoka University, Shizuoka, Japan

**Keywords:** endosymbiosis, cytoplasmic incompatibility, reproductive manipulation, horizontal gene transfer, suppressor

## Abstract

Cytoplasmic incompatibility (CI) drives maternally transmitted endosymbionts such as *Wolbachia* through insect populations by inducing embryonic mortality when infected males fertilize uninfected females. CI is controlled by *Wolbachia cif* operons that are categorized into multiple phylogenetic types. CI strength is further shaped by poorly understood host factors, including development and genetic background. To study the strength of CI across different host species, we genotyped a Japanese field population of *Liriomyza sativae*. By uncovering paternal transmission of *Wolbachia* genic elements, we collected strong evidence of horizontal genome transfer, including Type I and Type V *cif* operons, from *Wolbachia* into the nuclear genome of *L. sativae*. We established a transinfection of *w*Ltri in *L. sativae*, a *Wolbachia* variant that induces strong CI in *Liriomyza trifolii*. No CI was observed in both intraspecific and interspecific reciprocal crosses with *L. trifolii,* suggesting that both uninfected females and infected males of *L. sativae* completely suppress *w*Ltri-mediated CI. Our results raise the appealing hypothesis that host suppression of *Wolbachia-*induced CI might evolve owing to horizontal transfer of *cif* operons into the host nuclear genome.

## 1. Introduction

Maternally transmitted symbiotic bacteria, such as *Wolbachia*, commonly infect arthropods and spread through host populations by altering host reproduction [1–3]. Symbiont-mediated reproductive phenotypes include parthenogenesis, male killing, feminization, sex allocation distortion and cytoplasmic incompatibility (CI) [1,4–8]. CI causes embryonic lethality when infected males fertilize uninfected females [9,10]. Embryonic survival is rescued when females are infected*,* facilitating the spread of these maternally transmitted symbionts. Cytological studies have shown that CI-induced embryonic lethality is associated with inappropriate paternal chromatin condensation and segregation during the first mitotic cycle [11–13].

The genic factors of *Wolbachia* that control CI induction and rescue have been identified in several CI-inducing *Wolbachia* variants*,* including *w*Pip and *w*Mel [14,15]. The CI factor (*cif*) genes *cifA* and *cifB* often reside within the *Wolbachia* prophage regions and appear to form operons [14–16]. To induce CI, CifB effectors are necessary in infected males, whereas CifA effectors also contribute in certain systems but not in all. To rescue CI, CifA proteins are necessary in infected females in all systems studied thus far [16–19]. Phylogenetic reconstruction places *cif* operons into multiple types [20]. Several mechanistic models, including the host modification model (HM model) and toxin-antidote model (TA model) have been proposed to explain how Cif effectors regulate CI [21–23]. The HM model postulates that Cif effectors modify paternal chromosomes during spermatogenesis in infected males, and that CI rescue is achieved by CifA-mediated modification of maternal chromosomes during oogenesis. By contrast, the TA model proposes that CifB effectors function as toxins and are paternally transmitted to fertilized eggs to induce CI. Here, rescue of CI is achieved by cognate binding of CifA to CifB. These (and other) mechanistic models of CI are still being further developed as more evidence is gathered, including evidence of the cellular targets of CifB.

Theoretical studies indicate that selection on arthropod hosts can favour the evolution of host suppressors that reduce the strength of CI [24–28]. Experimental studies provide support for this prediction by showing that host species and genotype strongly determine CI strength [29–33]. These inter- and intraspecific host effects are predicted to contribute to variation in *Wolbachia* infection frequency and persistence across host populations and species. Yet, the genetic basis of CI suppression in arthropods remains unknown, with some evidence for a polygenic architecture in *Tetranychus* spider mites [30–36].

Endosymbiotic *Wolbachia* further shape host genetics by horizontal gene transfer (HGT). Horizontally acquired *Wolbachia* genetic elements have been documented in numerous species of nematodes, insects and isopods [34–40]. The horizontally transferred genetic elements of *Wolbachia* range in size from single genes to entire genomes [41]. Although the exact transfer mechanisms remain poorly understood, infection of *Wolbachia* in the germline cells of arthropod hosts is believed to facilitate heritable HGT [42]. Additionally, some transferred genetic elements from *Wolbachia* include apparent prophage regions [36,43]. Many *Wolbachia*-derived genes in arthropod genomes exhibit signs of pseudogenization, suggesting that these genes may have become non-functional [41,44,45]. However, studies have inferred that nuclear *Wolbachia* inserts might continue to shape host reproduction, as observed in the isopod *Armadillidium vulgare* [46]. Furthermore, these transferred genes are transcribed in a life stage- or tissue-specific manner [47,48], though their transcription levels are generally considered very low [41,44,45].

*Liriomyza trifolii* and its close relative *Liriomyza sativae* (Diptera: Agromyzidae) are important cosmopolitan agricultural pests with overlapping host plant ranges [49]. Invasive populations of *L. trifolii* have been reported in Japan from 1990 [50] and are infected with CI-inducing *Wolbachia* [51,52]. By contrast, although *Wolbachia* infection has been reported for *L. sativae* in the United States, Vietnam, Timor-Leste and Australia [53], invasive populations of *L. sativae* in Japan do not seem to carry *Wolbachia* (0 out of 118 flies in four prefectures appeared infected [52]). It is currently unclear whether *Wolbachia* that infect *L. sativae* outside of Japan are able to mediate CI. In Japan, *L. sativae* and *L. trifolii* display similar phenologies and can be found in sympatry [54]. Based on controlled crosses using laboratory populations, interspecific sterile hybrids can be produced [55,56]. In the current study, we examined the apparent uninfected state of Japanese *L. sativae* more carefully and gathered strong evidence that flies from an Umegashima field population carry horizontally acquired *Wolbachia* genetic elements in their nuclear genome. We established a *Wolbachia* transinfection in *L. sativae* and quantified CI strength using an exhaustive combinatorial genetic cross design. We did not observe demonstrable CI in *L. sativae,* an observation that could be linked to the presence and transcription of *cif* operons in the nuclear *Wolbachia* insert.

## 2. Results

### 2.1. Evidence for horizontal gene transfer from *Wolbachia* to *Liriomyza sativae*

**Figure 1.**
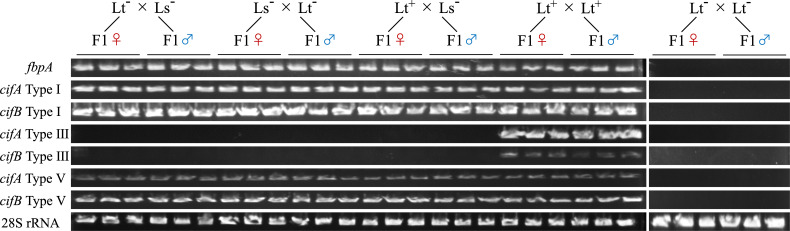
Type I and V *cif* operons and *fbpA* of *Liriomyza sativae* are paternally transmitted to interspecific hybrid males and females. Type III *cifA* and *cifB* do not display paternal transmission in *L. sativae,* suggesting that this operon is not encoded by the nuclear *Wolbachia* insert. Cross types are indicated by male × female. Lt^-^Lt^-^ is a negative control, while Lt^+^Lt^+^ was used as a positive control. 28S rRNA was used to control for DNA extraction. Amplicon size was approximately 500 bp for *fbpA,* approximately 300 bp for Type I *cifA*, approximately 500 bp for Type III *cifA*, approximately 150 bp for Type V *cifA*, approximately 750 bp for Type I *cifB,* approximately 120 bp for Type III *cifB* and approximately 320 bp for Type V *cifB*. For each cross type, three hybrid males and three hybrid females were tested.

*Liriomyza* populations were classified based on morphological characteristics of the male genitalia and maximum-likelihood phylogenetic reconstruction using *COI* and 28S rRNA sequences (electronic supplementary material, figure S1). Together, these taxonomic analyses show that *Liriomyza* flies collected in Shizuoka prefecture, Hamamatsu, belonged to *L. trifolii*, whereas *Liriomyza* flies from Umegashima belonged to *L. sativae*. Using our multi-marker diagnostic test, *L. trifolii* was considered as a *Wolbachia-*infected strain (electronic supplementary material, table S1 and figure S2). By contrast, the *L. sativae* Umegashima strain tested negative for all *Wolbachia* multilocus sequence typing (MLST) markers except for *fbpA* (electronic supplementary material, table S1, figures S2 and S3). A comparison of the sequences of the *fbpA* fragment between *L. trifolii* and *L. sativae* showed a 97% match (328/339 bp, electronic supplementary material). Using the fragmented *w*Ltri *Wolbachia* genome as a reference [57], we tested whether the genes adjacent to *fbpA* (*clpA* and *pyrD*) could also be detected in the *L. sativae* Umegashima strain (electronic supplementary material, figure S4). After successful polymerase chain reaction (PCR) amplification, Sanger sequencing showed that the *pyrD* and *clpA* genic fragments of *L. sativae* and *L. trifolii* had a 98% (866/881 bp) and 94% (1158/1231 bp) match, respectively (electronic supplementary material). Three non-synonymous substitutions were observed in the *fbpA* fragment between *w*Ltri and *L. sativae* (K5R S8R and K108R, numbering based on amplicon fragment). By contrast, a mutation in *pyrD* resulted in a premature stop codon, and multiple frameshift mutations were observed in *clpA* (electronic supplementary material), suggesting that these two latter genes might have undergone pseudogenization in *L. sativae*. Together, the Sanger read data suggest that a large genomic fragment of *Wolbachia* might have been incorporated into the nuclear genome of *L. sativae*. To provide more evidence for this hypothesis, we performed antibiotic curing of both *L. sativae* and *L. trifolii*. Whereas antibiotic curing resulted in a failure of PCR amplification of all *Wolbachia* MLST markers for *L. trifolii*, we still detected *fbpA* in the cured *L. sativae* line, consistent with our HGT hypothesis (electronic supplementary material, figures S2 and S3). Finally, to confirm the physical incorporation of *Wolbachia* genetic elements in the *L. sativae* nuclear genome, we tested for paternal transmission of *fbpA* from *L. sativae*. We crossed *L. sativae* males to cured *L. trifolii* females*,* generating interspecific hybrids. Our genotyping efforts revealed the presence of *fbpA* in both male and female hybrids (figure 1, three hybrid males and three hybrid females per cross type), firmly demonstrating paternal transmission and excluding the possibility that *fbpA* resides on a cytoplasmic *Wolbachia* symbiont.

### 2.2. Nuclear *Wolbachia* insert in *Liriomyza sativae* encodes *cif* operons

Based on the current reference genome assembly, *w*Ltri *Wolbachia* encode three *cif* operons, belonging to Type I, III and V [57]. We designed primers targeting divergent regions to specifically amplify these three types (electronic supplementary material, table S2). We detected both *cifA* and *cifB* genic fragments from the Type I and Type V operons in *L. sativae* from Umegashima, using infected *L. trifolii* as a control (electronic supplementary material, figure S5). In our interspecific crosses, Type I and V *cif* genes were detected regardless of offspring sex or parental combination (figure 1). For the Type I *cif* operon, whereas synonymous substitutions occurred in *cifA,* Sanger sequence data revealed seven non-synonymous and one synonymous substitutions within the predicted deubiquitylating enzyme (DUB; Ulp1) domain of *cifB* (1aa−125aa; electronic supplementary material, associated Dryad repository). For the Type V *cif* operon, the sequences of the *cifA* and *cifB* fragments were identical between *L. trifolii* and *L. sativae*. However, we did not detect the Type III *cif* operon in *L. sativae* using our diagnostic set-up (figure 1; electronic supplementary material, figure S5). Laterally acquired Type I and V *cifA* were transcribed in female *L. sativae* adults (electronic supplementary material, figure S6). PCR amplification further confirmed that the laterally acquired Type I and V *cif* genes remained adjacent (in an operon-like arrangement) following the incorporation into the *L. sativae* genome (electronic supplementary material, figure S7).

### 2.3. Loss of *w*Ltri-induced cytoplasmic incompatibility in a *Liriomyza sativae* genetic background

**Figure 2.**
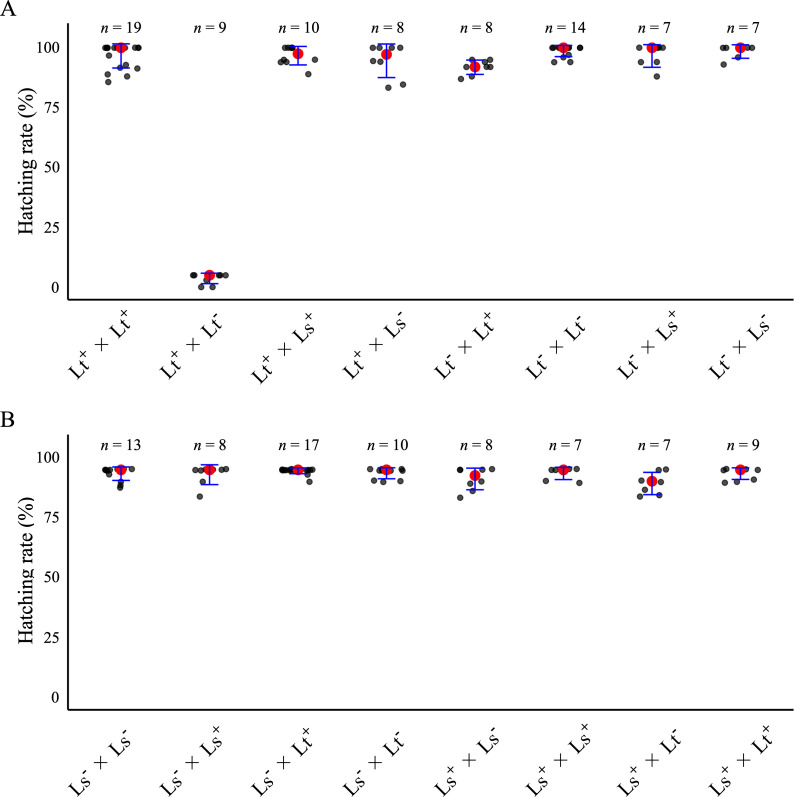
*w*Ltri *Wolbachia* do not induce cytoplasmic incompatibility against a *Liriomyza sativae* genetic background. Black dots represent data from individual female flies, red dots are averages and the blue lines are error bars. Cross types are indicated by male × female. The number of replications is shown above each cross type. Panel A shows the crosses with *Liriomyza trifolii* males, whereas panel B shows the crosses with *L. sativae* males.

The apparent horizontal acquisition of *cif* operons in *L. sativae* raises the question whether and how CI might be manifested in this leaf-mining fly population. We first confirmed that *w*Ltri *Wolbachia* induce strong CI in *L. trifolii* [57]. When infected *L. trifolii* males were crossed to uninfected *L. trifolii* females, egg hatching rate was significantly reduced (2.6%, with the compatible control cross at 95.0%, Steel’s multiple comparison test, *p* < 0.05; figure 2A). Microinjection successfully established a *w*Ltri *Wolbachia* transinfection in *L. sativae*. Out of the 20 pupae injected, 12 successfully emerged as adults. Of these, 10 individuals were confirmed to be infected with *w*Ltri based on our diagnostic PCR tests. Control *L. sativae* individuals that were injected with distilled water did not display a *Wolbachia* infection. *Wolbachia* transmission to the F_2_ generation was 100%, and infection remained fixed in the F_3_ generation (all 85 tested individuals tested positive; electronic supplementary material, figure S8), hereby confirming stable maternal transmission of *w*Ltri in transinfected *L. sativae*. We designate transinfected *L. sativae* as *L. sativae* W+.

For *L. sativae,* compatible control crosses resulted in a hatching rate of 97.6%. In the two interspecific CI crosses between *L. trifolii* and *L. sativae* (*L. trifolii* W+ male × *L. sativae* W− female, *L. sativae* W+ male × *L. trifolii* W− female), hatching rate did not significantly differ from that of compatible crosses (Steel’s multiple comparison test, *p* > 0.05; figure 2A,B; detailed data are available in the electronic supplementary material, table S3). Therefore, we were unable to demonstrate CI in both interspecific crosses. In addition to hatching rate, sex ratio also did not differ significantly between the intra- and interspecific combinations (Kruskal–Wallis test, *p* > 0.05).

**Figure 3.**
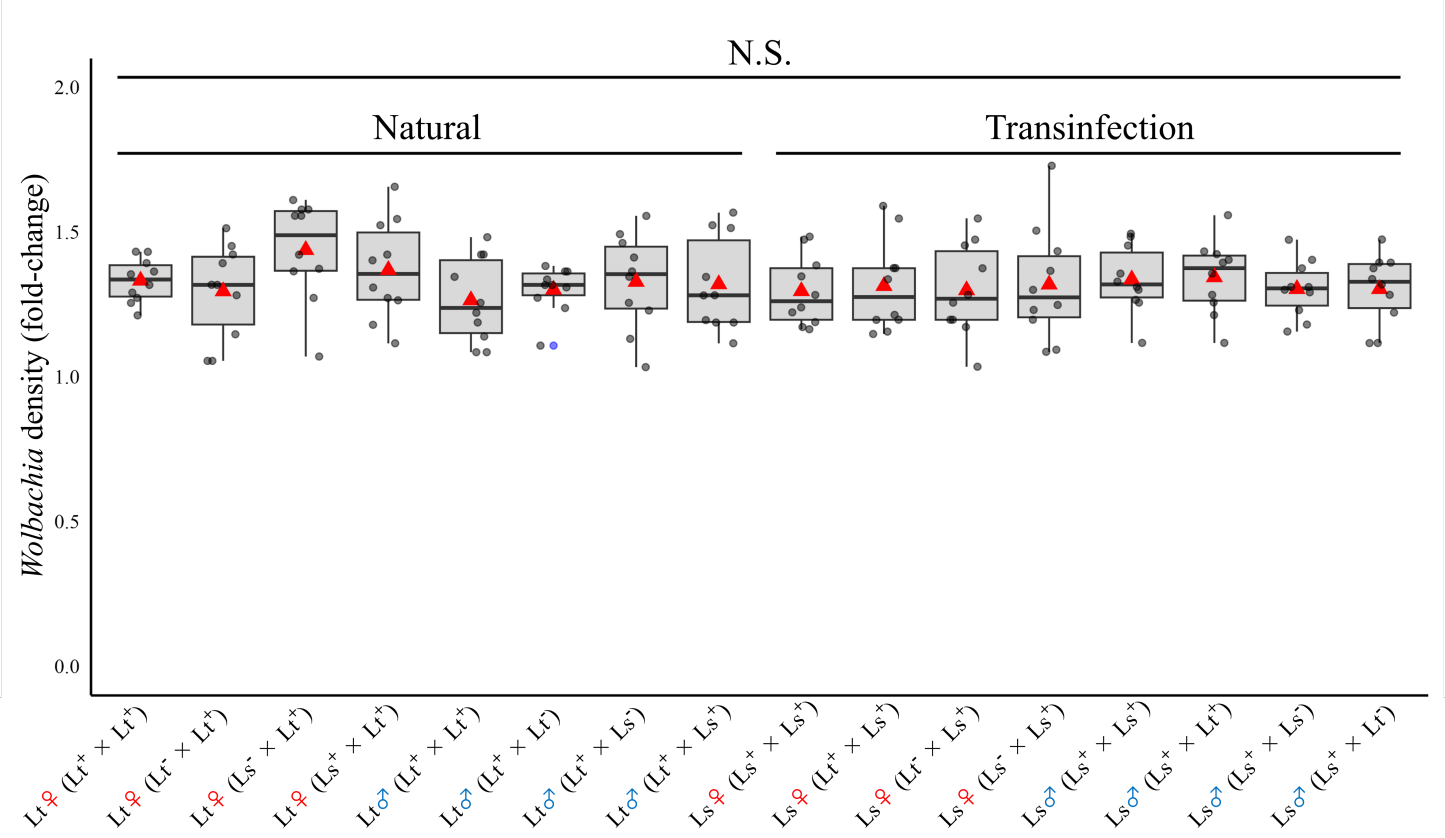
*Wolbachia* reach similar densities in our transinfected *Liriomyza sativae* and infected *Liriomyza trifolii* lines. *Wolbachia* density of each parent that was infected with *Wolbachia* used in the crossing experiment. The data obtained from each individual is shown in a jitter plot and box-and-whisker plot. The grey dots represent individual data, the red triangle depicts the mean and the blue dots depict outliers. *n* = 10 for all experiment plots. Fly genotypes are delineated below. For example, Lt male (Lt^+^ × Lt^+^) represents the *Wolbachia* density of Lt^+^ male individuals used in the Lt^+^ male × Lt^+^ female crossing experiments.

We confirmed the *Wolbachia* infection status of the parents used in all genetic crosses (electronic supplementary material, figure S8). There was no significant difference in the *Wolbachia* density in *L. trifolii* W+ and *L*. *sativae* W+ (transinfected strain) (one-way analysis of variance (ANOVA), *p* = 0.74；figure 3). Therefore, *w*Ltri *Wolbachia* density in transinfected *L. sativae* was comparable to the natural *Wolbachia* infection in *L. trifolii*. We further quantified the efficacy of *Wolbachia* maternal transmission in these interspecific crosses. In those crosses with an infected mother, the F_1_ generation of interspecific hybrids was infected at a frequency of 90–100% (electronic supplementary material, table S3).

Finally, we verified whether our interspecific F_1_ hybrids were sterile, as previous studies suggested [58]. Egg hatching rate for (Lt^+^ × Ls^−^) hybrid F_1_ males crossed with Lt^+^ females was 90% (*n* = 2). For Lt^+^ males crossed with (Ls^−^ × Lt^+^) hybrid F_1_ females, egg hatching rate was 95.2% (*n* = 2). Finally, egg hatching rate for (Ls^−^ × Lt^+^) hybrid F_1_ males crossed with Ls^−^ females was 87.5% (*n* = 3). Collectively, these findings indicate that the interspecific F_1_ hybrids between our *L. sativae* and *L. trifolii* strains are able to reproduce.

## 3. Discussion

In the current study, evidence was collected that a single or multiple *Wolbachia* genomic fragment(s) carrying *cif* operons are physically incorporated into the nuclear genome of Japanese *L. sativae*. Considering host species may acquire *Wolbachia* symbionts through horizontal transfer from sympatric closely interacting insects and through interspecific hybridization followed by introgression [58–62], we hypothesize that *w*Ltri *Wolbachia* of *L. trifolii* might be the donor variant for the *Wolbachia* nuclear insert in Japanese *L. sativae*. In *L. sativae,* these genes of *Wolbachia* origin were detected after antibiotic treatment and were paternally transmitted into interspecific hybrids. Current genomic data show that *w*Ltri *Wolbachia* encode Type I, III and V *cif* operons in Japanese *L. trifolii* [57]. For our interspecific hybrids, whereas Type I and V *cif* operons followed the expected paternal transmission mode with *L. sativae*, the Type III *cif* operon was only maternally transmitted from infected *L. trifolii* females. This suggests that, if *w*Ltri is indeed the donor variant, the Type III *cif* operon might not have been horizontally transferred into *L. sativae*, raising questions on the size and number of laterally transferred genomic region(s). Our PCR tests confirmed that for both Type I and V, *cif* genes remain adjacent within the *L. sativae* genome following the HGT event(s). Whether *w*Ltri, or *w*Ltri-like, is the original cytoplasmic *Wolbachia* donor variant requires formal validation. Although HGT from microorganisms into insect recipients is pervasive, the exact mechanisms that underpin these transfer events remain poorly understood [63,64]. In *Wolbachia*, *cif* operons can be encoded by plasmids and are often located within or near prophage regions and insertion sequence transposable elements, associations that may facilitate HGT [65–67]. Genome sequencing and characterization of Japanese *L. sativae* would address several of these outstanding questions. In *Drosophila ananassae,* gene duplication events of *cifA/B* operons occurred following horizontal genome transfer of *Wolbachia* [68]. Our observation that paternal transmission of genes of *Wolbachia* origin occurs in both *Liriomyza* males and females suggest that the nuclear insert(s) do not reside on the Y chromosome [69]. In *D. ananassae,* a nuclear integration event occurred of a single *w*Ana variant, followed by a geographical spread [70]. Future work should be directed at elucidating the spread of the *Wolbachia* insert(s) in *L. sativae* populations.

*w*Ltri *Wolbachia* do not induce demonstrable CI when infected *L. trifolii* males are crossed to uninfected *L. sativae* females. Moreover, upon *w*Ltri transinfection of *L. sativae*, CI could also not be demonstrated in intraspecific CI crosses, despite *w*Ltri reaching equal density levels as in naturally infected *L. trifolii*. Whereas some studies have also found an unaltered *Wolbachia* density between transinfection and natural infection, other cases report increased density levels, showing a complex interplay between host and *Wolbachia* genetics [71,72].

It is tempting to speculate that the horizontal acquisition of *Wolbachia cif* operons in *L. sativae* might be associated with the apparent suppression of CI in this host background. With cytoplasmic *Wolbachia,* CI is rescued by CifA effectors in infected females [73]. For *L. sativae* females*,* the observed Type I and V *cifA* transcription from the nuclear *Wolbachia* insert could explain the apparent absence of CI when crossed to infected *L. trifolii* males. Loss of *w*Ltri-mediated CI in crosses with infected *L. sativae* males could be associated with a divergent production and localization of CifA proteins during spermatogenesis. Alternatively, other host factors, independent of the *Wolbachia* nuclear insert, could modulate CI strength, as evidenced by previous studies [29,31,65]. These host factors could, for instance, suppress CI strength by either preventing CifB entry into sperm nuclei or by target-site mutations that inhibit chromatin–CifB interaction.

CI suppression has been confirmed in our *L. sativae* Umegashima strain, raising the question of whether CI can be expressed in other *L. sativae* populations. *Wolbachia*-infected flies have been reported in *L. sativae* populations in the USA, Vietnam, Timor-Leste and Australia [67]. It would be interesting for future work to examine the presence of *Wolbachia* nuclear insert(s) and whether symbiotic *Wolbachia* induce CI in these populations.

The high maternal transmission rate of *Wolbachia* into interspecific F_1_ hybrids suggests that *Wolbachia* may spread between sympatric populations of *L. sativae* and *L. trifolii*. In previous studies, F_1_ hybrids of *L. trifolii* and *L. sativae* in laboratory crosses followed Haldane’s rule, with only sterile females hatching [52,53]. In this study, we obtained fertile F_1_ individuals of both sexes and crossed these F_1_ hybrids with both parental species via backcrossing, further arguing that *Wolbachia* could spread across *Liriomyza* species boundaries by species hybridization [55,56]. Nevertheless, CI suppressors within *L. sativae* may prevent the further spread of introgressed *w*Ltri in Japanese *L. sativae*.

## 4. Material and methods

### 4.1. *Liriomyza* fly strains

The *L. trifolii* strain was collected from Hamamatsu City, Shizuoka Prefecture, Japan, in 1991. *Liriomyza sativae* was obtained from the leaves of pumpkin (*Cucurbita maxima* Duchesne ex Lam.) collected from Umegashima, Shizuoka Prefecture, Japan, in 2018. Both strains were raised on the quail bean variety *Phaseolus vulgaris* Linnaeus. The insects were maintained at 25°C in an illuminated incubator (MLR-351H, SANYO, Japan) with a 16 L : 8 D cycle. Both fly species were identified based on differences in the length of the mesophallus among the male genitalia [50]. Genital morphology terminology was based on [49].

### 4.2. DNA extraction and molecular species identification

Genomic DNA from whole bodies were individually extracted using 30 μl sodium chloride–Tris–EDTA buffer {5N NaCl, 500 mM EDTA [pH 8. 0], and 1 M Tris-HCl [pH 8. 0]} and 2 μl proteinase K by incubation at 56°C for 2 h and at 99°C for 3 min. DNA extracts were eluted in a final volume of 30 μl and stored at −20°C. To confirm the species identification, the *COI* and 28S rRNA PCR products of a subset of individuals were Sanger sequenced. Primers are shown in the electronic supplementary material, table S1, and all sequence GenBank accession numbers are listed in the electronic supplementary material, table S4. To confirm *Liriomyza* species, we used LCO1490-HCO2198 primer [74] and 28S rRNA primers [75,76]. PCR reactions were prepared using 0.1 μl of TaKaRa Ex Taq (Takara Bio, Japan) with a final concentration of 1 μM of each primer, 14.1 μl of distilled water, 2.0 μl of 10×Ex Taq buffer, 1.6 μl of 2.5 mM deoxynucleotide triphosphates mixture and 0.6 μl template DNA, to a final reaction volume of 20 μl. Reactions were run on a TaKaRa PCR Thermal Cycler Dice (TP600, Takara Bio) for 10 min at 95°C, followed by 35 cycles of 94°C for 10 s, 52°C for 30 s and 72°C for 60 s, and then an extension reaction step at 72°C for 2 min. PCR products were electrophoresed on a 1.6% agarose gel and visualized using Midori Green staining (Nippon Genetics Co., Japan).

### 4.3. Phylogenetic reconstruction

Phylogenetic trees were constructed using *COI* and 28S rRNA genes of *L. trifolii* and *L. sativae* obtained by sequencing. In addition, Blast searches were performed, and *COI* and 28S rRNA genes of other *Liriomyza* species were collected and included for phylogenetic analysis. Species names and accession numbers are listed in the electronic supplementary material, table S4. Alignments were constructed with MEGA X [77] and MUSCLE [78]. Phylogeny was inferred by using the maximum likelihood method and general time reversible model [79]. We generated a bootstrap consensus tree inferred from 1000 replicates [80]. Initial trees for the heuristic search were obtained automatically by applying Neighbor-Join and BioNJ algorithms to a matrix of pairwise distances estimated using the maximum composite likelihood approach and then selecting the topology with superior log likelihood value. A discrete Gamma distribution was used to model evolutionary rate differences among sites (two categories (+G, parameter = 0.1734)). The rate variation model allowed for some sites to be evolutionarily invariable ([+I], 37.44% sites). This analysis involved 12 nucleotide sequences. There were a total of 1477 positions in the final dataset. These analyses were conducted using MEGA X software.

### 4.4. Diagnostic assays of *Wolbachia* infection

To test for *Wolbachia* infection, we used the *Wolbachia*-specific primers wsp-81F and wsp691R [81], MLST primers and a supergene alignment of 2079 nucleotides based on five housekeeping genes (*coxA*, *gatB*, *ftsZ*, *hcpA* and *fbpA;* [82]). PCR set-up was changed from the above protocol to an annealing temperature of 55°C. Primers rc28A(F) and 28C(R) amplify a 28S fragment of the host and were used to control the DNA extraction procedures.

### 4.5. Detection of laterally transferred *Wolbachia* genic fragments

Functional analysis and visualization were performed by Proksee [83] and InterProScan [84] using a previously published fragmented *w*Ltri genome assembly [57]. Primers were generated by Primer3 Plus [85] for *clpA* and *pyrD*, the predicted neighbours of *fbpA*. Primers were also created for *fbpA*, which amplified a 2200 bp fragment. The *fbpA*, *cifA* and *cifB* genes in *L. trifolii*, *L. sativae* and F_1_ hybrids were confirmed by PCR. *cif* primers were created with Primer3 Plus using *cif* sequence data (Type I, Type III and Type V) of a previously published fragmented *w*Ltri genome assembly [57].

PCR reaction mixtures included 25 µl of KOD One^®^ PCR Master Mix -Blue- (Takara), 21 µl of distilled water, 1.5 µl each of primers clpAF and clpAR, pyrDF and pyrDR, fbpA+hF and fbpA+hR, fbpAF and fbpAR, cifAF and cifAR or cifBF and cifBR, and 1.0 µl of DNA extract. Primers rc28A(F) and 28C(R) were used as positive controls. The PCR protocol was as follows: initial denaturation at 98°C for 2 min, followed by 30 cycles of 98°C for 10 s, 53°C for 5 s and 68°C for 3 s, with a final extension at 68°C for 10 min. Amplified PCR products were electrophoresed on a 1% agarose gel. Sequence data were contracted to Fasmac, Inc. (Kanagaea, Japan).

Based on the obtained sequences, sequence alignment for each gene was performed using ClustalW to identify conserved regions and potential sequence variations. Subsequently, amino acid sequences were predicted using the ExPASy Translate Tool [86], ensuring accurate conversion of nucleotide sequences into their corresponding protein sequences. Functional annotations of the predicted amino acid sequences were conducted using InterProScan 5 [84,87].

### 4.6. *cifA* transcription and *cif* operon structure in uninfected *Liriomyza sativae*

To determine whether the horizontally acquired *cifA* gene is transcribed in female *L. sativae* adults, RNA was extracted from a single female adult of *L. sativae* and *L. trifolii* 24 h after emergence using an RNeasy Plus Micro Kit (QIAGEN, Hilden, Germany). cDNA was synthesized from total RNA using the PrimeScript^®^ II 1st Strand cDNA Synthesis Kit (Takara Bio Inc., Shiga, Japan). Based on *w*Ltri genomic data [57], *cif* coding regions were predicted using ORFfinder and GenScan [88]. Primers targeting these regions were designed using Primer3 Plus ([85]; electronic supplementary material, table S2). For Type I *cifA* and Type V *cifA*, reverse transcription-PCR (RT-PCR) was performed on cDNA of *L. sativae* and *L. trifolii* using the KOD One^®^ PCR Master Mix -Blue- (Toyobo, Osaka, Japan). For a positive control, primers targeting *Ef1a* were used, whereas for the negative control, PCR was performed on RNA samples prior to RT. PCR cycling conditions were as follows: an initial denaturation at 98°C for 2 min, followed by five cycles of 98°C for 10 s, 60°C for 10 s and 68°C for 10 s; five cycles of 98°C for 10 s, 56°C for 10 s and 68°C for 10 s; and 20 cycles of 98°C for 10 s, 52°C for 10 s and 68°C for 10 s. A final elongation step was carried out at 68°C for 10 min. Amplified RT-PCR products were electrophoresed on a 1% agarose gel.

To determine whether horizontally acquired *cifA* and *cifB* retained their operon structure in uninfected *L. sativae*, primers were designed to target the 3′ end of *cifA* and the 5′ end of *cifB* for both Type I and Type V. Primer design was based on *w*Ltri genomic data [57] and was conducted using Primer3 Plus ([85]; electronic supplementary material, table S2). PCR was performed in reaction mixtures containing 25 µl of KOD One PCR Master Mix -Blue- (Takara Bio Inc.), 21 µl of distilled water, 1.5 µl each of primers Type I cif1oF and Type I cif1oR, Type V cif5oF and Type V cif5oR and 1.0 µl of DNA extract. The rc28A(F) and 28C(R) primers, which target the 28S rRNA gene, were used as positive controls. PCR conditions were as follows: an initial denaturation at 98°C for 2 min, followed by 30 cycles of 98°C for 10 s, 58°C for 5 s and 68°C for 3 s, with a final extension at 68°C for 10 min. Amplified PCR products were electrophoresed on a 1% agarose gel.

### 4.7. Quantitative polymerase chain reaction

*Wolbachia* density levels were evaluated by quantitative PCR on a Thermal Cycler Dice Real-Time System II (TP 970; Takara). The total reaction volume of 25 µl contained 12.5 µl TB Green Premix Ex Taq II (Takara), 8.5 µl sterile water, 1 µl each of forward and reverse primers and 2 µl template DNA. Cycling conditions were 98°C for 10 s, followed by 40 cycles of 98°C for 10 s and 55°C for 30 s. Relative *Wolbachia* density was calculated with the 2^−∆∆Ct^ method [89] by comparing the copy number of the *wsp* gene to that of the elongation factor 1α (Ef1a) gene [90] L.tri wsp-F and L.tri wsp-R. Ef1a primer was designed using NCBI Primer 3 Plus [91]; *Liriomyza* Ef1a-F and *Liriomyza* Ef1a-R. Ten replicates were established for each experimental plot (five biological replicates, each with at least two technical replicates for each gene). Statistical analysis of the obtained results was performed by means of ANOVA.

### 4.8. Antibiotic treatments

To cure *Wolbachia* from *L. trifolii*, a solution containing an antibiotic (tetracycline hydrochloride; FUJIFILM Wako Pure Chemical, Japan) and honey (50 mg ml^−1^) was prepared, adsorbed using cotton and fed into a 1.5 ml tube (RIKEN, Japan) together with pupae of *L. trifolii*. At 24 h after hatching, these adults were fed quail bean leaves sprayed with antibiotics and reared in an incubator (16 L : 8 D, 25°C) to lay their eggs. Tetracycline hydrochloride and mycoshield hydrate (Pfizer, USA) were diluted ×100 with distilled water and sprayed directly onto the quail bean leaves using a mist sprayer. The treated quail beans were placed in acrylic rearing containers (35 cm × 35 cm × 35 cm), with five plants per cage. To verify the effectiveness of the antibiotic curing, six individuals of a subsequent generation were randomly selected to determine whether they were infected with *Wolbachia* by PCR using *wsp* primers. Primers rc28A(F) and 28C(R) were used as positive controls.

### 4.9. *Wolbachia* transinfection in *Liriomyza sativae*

*Wolbachia* was not detected in *L. sativae* based on the PCR results. Therefore, we injected *w*Ltri into *L. sativae* pupae to generate *Wolbachia*-infected flies. Twenty adult *L. trifolii* were surface sterilized with ethanol and placed in 1.5 mm tubes, and after adding 300 μl of phosphate-buffered saline, the pupae were mashed. The *Wolbachia* solution was prepared by filtering the suspension through a 26Φ μm, 5.0 μm pore filter (Advantec, Tokyo, Japan). Subsequently, a calibrated micropipette (Drummond, Blue Mall, PA, USA) with a diameter of 0.26 mm was formed into a needle shape using a puller (PC-100, NARISHIGE, Tokyo, Japan) and set in a microinjection machine (MMO-4, NARISHIGE) to aspirate the *Wolbachia* solution. The needle was then inserted into the pupae of *L. sativae* from the centre to the lower abdomen, and the *Wolbachia* solution was injected.

This process was performed on 20 *L*. *sativae* individuals, and distilled water was used as a negative control. Unmated *L. sativae* injected with *Wolbachia* were paired for 24 h with one female and one male in the same test tube (7.5 cm long and 1.0 cm in diameter; Thermo Fisher Scientific, Waltham, MA, USA). Then, quail bean leaves were added to the rearing cages, which were prepared. The mated females were then released to lay their eggs and examined for *Wolbachia* infection. In addition, we fed the quail bean leaves to the next generation of F_1_ adults that hatched from each plot, allowed them to lay eggs and examined the eggs for *Wolbachia* infection. This method was repeated until the F_2_ generation. After confirming that *Wolbachia* was not eliminated from all individuals, this line was designated as the *L. sativae Wolbachia*-infected strain.

### 4.10. Intra- and interspecific cross design

Four strains were used in the crossing test: *Wolbachia*-infected and uninfected (antibiotic-treated) strains of *L. trifolii,* and *Wolbachia*-transinfected and uninfected strains of *L. sativae*. To avoid the direct effects of antibiotic treatment, *L. trifolii* uninfected individuals from the third generation onwards after antibiotic treatment were used. *Liriomyza sativae* infected individuals from the third generation onwards after injection of *w*Ltri were used. All 16 different combinations were performed in the cross experiments. All individuals used in the cross experiments were unmated, 24 h after emergence and 50% honey in water was provided as food. One unmated female and two unmated males were released into test tubes (7.5 cm long, 1.0 cm diameter; Thermo Fisher Scientific), and the tubes were plugged with cotton for 24 h. At this time, filter paper sections (1.0 cm × 1.0 cm: Advantec) soaked in a solution of 50% honey in water were provided as a food. After pairing, the females were released into the rearing containers. After 24 h, the quail bean leaves were removed from the rearing containers, the number of eggs laid was counted and the petioles were inserted into a conical flask filled with water. After egg hatching began, they were transferred to a square acrylic case (9.5 cm × 6.5 cm × 6.5 cm) with mesh, and the numbers of larvae, pupae and emerging adults were counted and sexed. After the experiments, we confirmed *Wolbachia* infection by diagnostic PCR using *wsp* primers in the parents and offspring. All individual rearing and crossing tests were conducted in a 16 L : 8 D 25°C incubator. Statistical analyses were conducted on the number of eggs laid, hatching rate, emergence rate, sex ratio and *Wolbachia* infection rate for F_1_. The reproductive ability of F_1_ males and females was assessed through backcrossing experiments.

### 4.11. Statistical analyses

In the crossbreeding experiments, the results of the Shapiro–Wilk test for normality revealed that not all data such as egg counts, hatching rates, emergence rates, sex ratios and infection rates of F_1_ individuals followed a normal distribution (*p* < 0.05).

Therefore, a non-parametric approach was adopted. Specifically, the Kruskal–Wallis test was employed to assess differences among groups, and Steel’s multiple comparison test was used to determine pairwise differences. Percentage data were logit-transformed prior to statistical analysis to prevent divergence to infinity or negative infinity, with 100% values treated as 99.999% and 0% values treated as 0.001%.

*Wolbachia* density was calculated based on the 2^−∆∆Ct^ method, referencing the ∆Ct values [89]. Subsequently, ANOVA was performed to compare groups.

Statistical analyses were conducted using R software [92,93] and R Studio [93]. For all statistical tests, a *p*-value of <0.05 was considered statistically significant.

## Data Availability

The authors confirm that the data supporting the findings of this study are available within the article or its electronic supplementary material [94]. All sequence data can be obtained by accessing NCBI using the accession numbers. The focal Liriomyza strains, and their availability, are described in the Material and Methods. This study did not generate any unique codes.
